# Improving the modelling of a multi-leaf collimator with tilted leaf sides used in radiotherapy

**DOI:** 10.1016/j.phro.2024.100543

**Published:** 2024-02-01

**Authors:** Mohammad Hussein, Agnes Angerud, Jordi Saez, Evelien Bogaert, Matthieu Lemire, Miriam Barry, Ileana Silvestre Patallo, David Shipley, Catharine H. Clark, Victor Hernandez

**Affiliations:** a Metrology for Medical Physics Centre, National Physical Laboratory, Teddington, UK; b RaySearch Laboratories AB, Stockholm, Sweden; c Department of Radiation Oncology, Hospital Clínic de Barcelona, Barcelona, Spain; d Department of Radiation Oncology, Ghent University Hospital, Belgium; e CIUSSS de l'Est-de-l'Île-de-Montréal, Quebec, Canada; f Medical Physics, University College London Hospital, London, UK; g Medical Physics and Bioengineering, University College London, London, UK; h Department of Medical Physics, Hospital Sant Joan de Reus, IISPV, Tarragona, Spain; i Universitat Rovira i Virgili, Tarragona, Spain

**Keywords:** MLC modelling, TPS modelling, TPS commissioning

## Abstract

**Background and purpose:**

Multi-leaf collimators (MLCs) with tilted leaf sides have a complex transmission behaviour that is not easily matched by radiotherapy treatment planning systems (TPSs). We sought to develop an MLC model that can accurately match test fields and clinically relevant plans at different centres.

**Materials and methods:**

Two new MLC models were developed and evaluated within a research version of a commercial TPS. Prototype I used adjusted-constant transmissions and Prototype II used variable transmissions at the tongue-and-groove and leaf-tip regions. Three different centres evaluated these prototypes for a tilted MLC and compared them with their initial MLC model using test fields and patient-specific quality-assurance measurements of clinically relevant plans. For the latter, gamma passing rates (GPR) at 2 %/2mm were recorded.

**Results:**

For the prototypes the same set of MLC parameters could be used at all centres, with only a slight adjustment of the offset parameter. For centres A and C, average GPR were >95 % and within 0.5 % GPR difference between the standard, and prototype models. In center B, prototypes I and II improved the agreement in clinically relevant plans, with an increase in GPR of 2.3 % ± 0.8 % and 3.0 ± 0.8 %, respectively.

**Conclusions:**

The prototype MLC models were either similar or superior to the initial MLC model, and simpler to configure because fewer trade-offs were required. Prototype I performed comparably to the more sophisticated Prototype II and its configuration can be easily standardized, which can be useful to reduce variability and improve safety in clinical practice.

## 1 Introduction

Robust fine-tuning of multi-leaf collimator (MLC) modelling parameters in radiotherapy Treatment Planning Systems (TPSs) is crucial for creating an optimal intensity-modulated radiotherapy (IMRT) beam model, particularly with the ever-increasing accuracy required for advancing techniques [1], [2].

MLCs can have complex geometries which vary between different manufacturer implementations [3], [4]. Key factors include: leaf thickness, the curvature of the leaf tip, the interlocking design between adjacent leaves, and the angle relative to the source. While the ideal dose calculation scenario is to Monte Carlo simulate the realistic geometry of MLCs, this is currently prohibitive within TPSs due to the computation time needed to simulate dynamically moving leaves. Instead, the key features of MLCs are implemented as different transmission regions through which the source model is ray traced. The complexity in the physical design of the MLC will dictate the number of transmission regions required to accurately mimic the fluence traversing through them, however this will be at a trade-off with computational resources. Limitations in accuracy make it necessary to deviate from physically realistic model parameters to compensate for calculation differences. This deviation can lead to a need for users to balance trade-offs in the tuning of multiple different parameters.

Furthermore, there is a lack of standardization of the procedures used for the configuration of MLC models. There are a variety of approaches ranging from simple test fields that aim to characterize key features of the MLC to clinically relevant plans which are used to iteratively fine-tune modelling parameters.

As a result of these factors, the quality of parameter tuning may depend on the experience of the physicist and the type of tests carried out. It has been shown in the literature that MLC parameter values used in the clinic vary between centres, which could lead to variations in the accuracy of dose calculation [5], [6].

Generically there are two main types of MLC, those that have a stepped tongue-and-groove (T&G) design and that have a tilted design. Stepped T&G MLCs are easier to model in TPSs than tilted MLC [3], [7]. Tilted MLCs are designed to reduce leaf transmission but this means that a progressive increase in the T&G shadowing is present, therefore more transmission regions may be needed to accurately mimic this behaviour. However, TPSs did not account for this effect and therefore larger trade-offs in optimisation of the model parameters are necessary. For this MLC type large variability in the final MLC parameters used by different centres has been shown in the literature [5], [8].

A novel methodology for robust convergence to an optimized MLC model has previously been proposed based on measurements of dynamic fields with a Farmer-type chamber [9], [10], [11] and has been shown to accurately characterize details of the MLC such as tongue-and-groove width and leaf-tip-width. Using this methodology, Saez et al described how to extract TPS parameters to reproduce the average doses of these fields for stepped T&G MLCs [11]. However, this methodology has not been tested for tilted MLCs.

Therefore, in this study we aimed to develop and evaluate two different MLC transmission maps, implemented as prototypes in a research version of a commercial TPS, to improve the modelling of a tilted MLC. Both prototypes aimed to match test fields typically used in MLC commissioning [12], [13] and the methodology proposed by Saez et al [11]. Additionally, the study investigated the feasibility of using a common set of MLC parameters for different centres and assessed the impact of these new MLC models using measured clinically relevant plans.

## 2 Materials and Methods

Three different institutions with Elekta linacs (two VersaHD and one Synergy) equipped with the Agility MLC (Elekta AB, Stockholm, Sweden) and the RayStation TPS (RaySearch Laboratories, Stockholm, Sweden) participated in this study, using the 6 MV flattened beam. The Agility MLC uses a flat slightly non-target pointing surface [14], [15]. The tilted leaf side is cut by the rounding of the leaf tip, reducing the maximum extension of the tongue-and-groove into the open region with a progressive increase of tongue-and-groove shadowing as far as 20 mm from the leaf tip end [7].

### 2.1 Sweeping gap, asynchronous sweeping gap tests and static tests

**Fig. 1:**
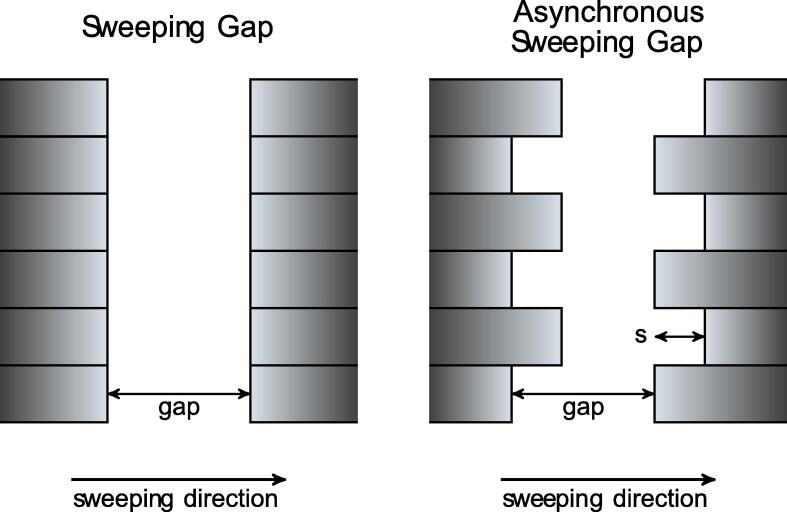
Schematic of the sweeping gap and asynchronous sweeping gap tests.

In sweeping gap (SG) tests, all leaves are placed at the same position, forming a rectangular aperture with a given gap, and leaves move at a constant speed from left to right. The asynchronous SG (aSG) tests, developed by Hernandez et al [9], are similar to SG, but adjacent leaves are offset a given distance, *s,* to expose a controlled amount of the leaf edges, thus leading to a non-rectangular aperture shape where the gap between leaf pairs remains constant (Fig. 1). The average dose per sweep was measured with a Farmer ion chamber at 10 cm depth in water, and 90 cm source-to-surface distance.

The same fields were calculated in the TPS using a virtual water phantom with the dose scored to a region-of-interest representing the collecting volume of the Farmer chamber.

Elekta provides a set of predesigned tests, known as the ExpressQA package [12], [13], as a tool to determine the MLC parameters in the TPS. The FOURL test was used to investigate tongue-and-groove modelling with film dosimetry performed using each centre’s film protocol (home-made MATLAB software, radiochromic.com, and FilmQA Pro, respectively). A y-profile from the FOURL film extracted at x = 4 cm is sensitive to the maximal tongue-and-groove shadowing away from the leaf tip.

### 2.2 Initial MLC model: Full transmission in the tongue-and-groove/leaf tip intersection

**Fig. 2:**
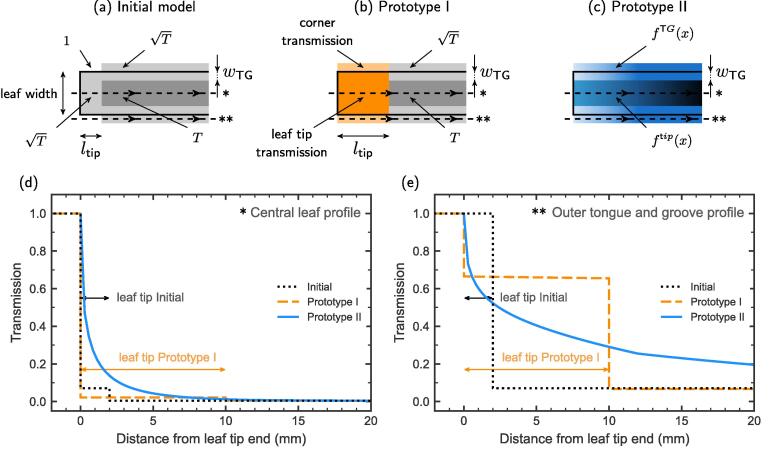
Visualisation of the TPS MLC models in the beams-eye-view. Transmission profiles under the centre of the leaf width and in the outer tongue-and-groove region as a function of the distance to the leaf tip end s. The areas under different profiles cannot be directly compared due to the different tongue-and-groove widths used in each model.

RayStation uses an MLC model with constant transmission regions; for version 12A and earlier, the leaf-tip and tongue-and-groove (TG) regions had transmission √T, where *T* is the average MLC transmission. The widths of these regions could be configured with the parameters “leaf-tip-width” and “tongue-and-groove”. The TG parameter indicated the width of the affected regions sticking both outwards and inwards with respect to the nominal leaf-width. Thus, the total width of the TG region considered by the MLC model at each leaf side was twice the TG parameter. The leaf-tip-width parameter indicated the distance from the leaf tip end in which an increased transmission $\sqrt{T}$ is assigned. No TG at the leaf tip is considered in this MLC model; therefore, a √T transmission was assigned below the leaf tip and full transmission (T = 1) was considered in the corner adjacent to the leaf tip. This MLC model is illustrated in Fig. 2a and the transmission profiles below the leaves and below the outer tongue-and-groove region are shown in Fig. 2d and 2e. Detailed information about the characteristics of the Elekta Agility MLC are described by Hernandez et al [7].

Three additional parameters (“offset”, “gain”, and “curvature”) were used to define the difference between the leaf positions used in dose calculation and the DICOM/UI positions of the leaf tips.

### 2.3 Prototype I: Adjusted transmission in the tongue-and-groove/leaf tip intersection

In Prototype I (Fig. 2b, 2d, 2e), the transmission of the leaf-tip and of the corner region were fitted to cross line profiles and to the measured aSG fields, as was the tongue-and-groove width. Adjusting the corner transmission to a value lower than 100 % produced a double linear fit in the calculated aSG curve instead of the constant plus linear fit described in Saez et al [11], allowing a better fit to the experimental aSG measurements.

### 2.4 Prototype II: Raytracing-adjusted transmission in the tongue-and-groove/leaf tip intersection

Prototype II (Fig. 2c, 2d, 2e) modelled the variable transmission from the leaf-tip towards the leaf base both below the leaf and over the tongue-and-groove region based on relative thickness of the known geometry and realistic shadowing. The relative thickness and transmission of two adjacent leaves were combined into interleaf and interdigitation transmission functions. The tongue-and-groove width was adjusted to fit the averaged measured aSG fields from the three centres.

### 2.5 Configuration of the MLC models

While each centre had arrived at different parameters in their initial MLC transmission models, both Prototype I and Prototype II had a common set of parameters with the exception of the MLC offset (Table 1). In centre A, the prototype MLC offset was tuned by finding the best match between the Farmer-measured asynchronous 20 mm gap test and dose calculation. Centre B tuned the offset based on optimizing the agreement between the measured central dose in Octavius4D and the calculated dose for five VMAT cases. In centre C, the offset was tuned by optimizing the difference between Farmer chamber measured dose in a low gradient region for 20 VMAT beams and the calculated dose. For simplicity, the gain and curvature were set to zero in both Prototypes for all centres.

**Table 1:** Summary with the MLC parameter values for the initial (Centre A, B, C), Prototype I and Prototype II MLC models.

**Parameter**	**Centre A**	**Centre B**	**Centre C**	**Prototype I**	**Prototype II**
Tongue-and-groove (cm)	0.05	0.07	0.1	0.09	0.123
Leaf tip width (cm)	0.46	0.25	0.6	1	–
MLC transmission	0.6 %	0.55 %	0.4 %	0.45 %	0.45 %
Offset (cm)	−0.012	−0.005	−0.02	−0.006 to 0.015	−0.077 to −0.093
Gain	0.0037	0.0007	0.008	0	0
Curvature (1/cm)	0.0001	−0.000015	0	0	0
Leaf tip transmission	√T			2.15 %	Function of distance to leaf tip end
Tongue-and-groove transmission	√T			√T	Function of distance to leaf tip end
Corner transmission	100 %			66.5 %	Function of distance to leaf tip end

### 2.6 Evaluation with clinically relevant plans

A set of clinically relevant plans were selected at each centre and with the aim to cover a wide range of treatment sites and target sizes, highly modulated plans were selected to challenge the different MLC models. Supplementary Figure S1 shows examples of different types of treatment plans included. Each centre measured and evaluated computed dose with their PSQA type devices and software. All patient plans were anonymous and recalculations with the prototype MLC models were performed on phantom datasets. Therefore no identifiable data or images were used and no further institutional approvals were required.

In centre A, two vertebra SBRT cases were measured with EBT3 radiochromic film within a solid water phantom: one plan with standard modulation and one with an intentionally high number of MUs (surrogate for modulation). A dual-arc head and neck VMAT plan and a single-arc prostate VMAT plan were measured using the PTW OctaviusII-729 detector array. A single isocentre three-metastasis stereotactic radiosurgery (SRS) case with four coplanar arcs was measured on the CIRS STEEV anthropomorphic phantom with EBT-XD radiochromic film in an axial and a sagittal plane. Alanine pellets [16] were used to measure the dose inside the largest GTV and in the brainstem region. Finally, a spine SBRT plan was made on a CIRS Atom phantom, in which the thorax-abdomen region was adapted in-house to allow for measurement of an axial EBT3 film and three alanine pellets through a region mimicking the T12 vertebra. Dose calculations were made with 1 mm dose grid spacing for the SBRT and SRS cases, otherwise 2 mm was used.

Analysis of radiochromic film and PTW Detector Array measurements was made using γ dose difference relative to prescription dose and 20 % lower dose threshold. An in-house software developed in MATLAB version 2020b (The MathWorks Inc.) was used for the analysis. Also mean γ was recorded.

In centre B, fifteen VMAT plans were chosen out of 142 clinical plans created over a six-month period. The plans had varying degree of complexity but all with unusually large tongue-and-groove exposure, they were all evaluated and found to be clinically representative. The plans covered paravertebral oligometastasis (N = 2), brain metastastasis (N = 1), prostate (N = 3), *para*-aortic pelvic lymph nodes (N = 1), oesophagus (N = 2) and head and neck (oro- and hypopharynx, larynx, oral cavity) (N = 6) plans.

The Octavius4D phantom with the Octavius 1500 array were used for measurements. The PTW Verisoft v8.0 software was used to reconstruct a measured 3D dose and perform 3D gamma evaluation between reconstructed dose and TPS dose. A correction for daily output was applied. Gamma dose difference was relative to calculated maximum dose and dose threshold was 10 % of the maximum dose. Gamma calculation used 2nd and 3rd pass filter, to avoid false positive results caused by the resolution of the detector [17].

In centre C, ten clinical VMAT plans were selected. Five were lung SBRT plans, three with a single PTV and two with two PTVs. Two whole brain plans with hippocampal sparing and simultaneous multiple metastasis boost were highly modulated and optimized with three complete VMAT arcs. The last three plans were head and neck, treated to either two or three simultaneous dose levels.

All plans were measured on the Sun Nuclear ArcCHECK diode array. Point dose measurements were also performed with a PTW N30001 Farmer chamber in a rectangular solid water phantom. 2-D analysis was performed using the Sun Nuclear SNC Patient software with 15 % lower dose threshold recording also mean γ.

## 3 Results

### 3.1 Sweeping gap and asynchronous sweeping gap tests

**Fig. 3:**
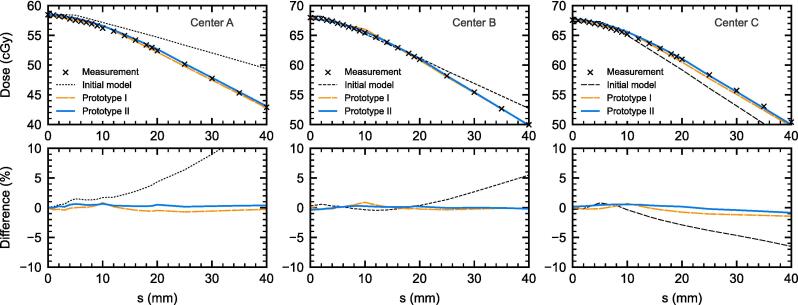
Measured SG and aSG tests compared with the dose calculations from the different MLC models. The dose values are shown in the top graphs, and percentage difference given in the bottom graphs.

For the 40 mm gap size, as shown in Fig. 3, the agreement between the measured (a)SG tests and the calculated dose by Prototypes I and II were within 1 % across all *s* values. However, the initial models diverged from the measurements at *s* > 10 mm for centres A and C, and *s* > 20 mm for centre B.

### 3.2 Static tests

**Fig. 4:**
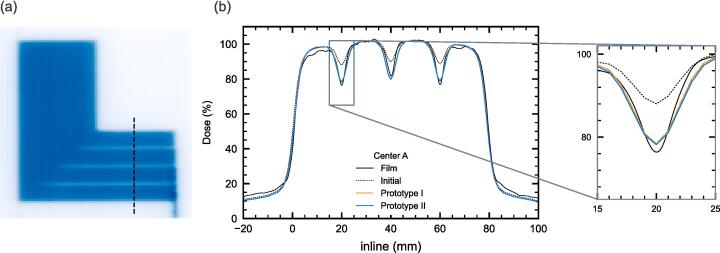
FOURL test results. A representative dose map measured with film dosimetry, in this case centre A, is shown in. Measured and calculated line dose profiles along the dashed line indicated in (a) are shown in (b).

For the FOURL test, a large tongue-and-groove width was used by Centre C, whereas the initial model obtained using the commissioning strategy of Centre A underestimated both the depth of the valleys and the overall shadowing effect of the tongue-and-groove far from the leaf tip (see Fig. 4 for centre A, Supplementary Figure S2 gives the plots for centres B and C).

### 3.3 Evaluation with clinically relevant plans

**Fig. 5:**
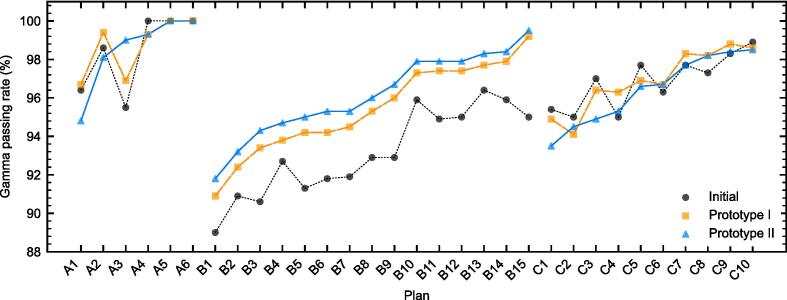
Summary of all the PSQA gamma passing rates according to each centre’s analysis for 2 %/2mm. The labels indicate centre and plan number.

The average GPR at 2 %/2mm were 98.4 % ± 2.0 % (initial model), 98.7 % ± 1.5 % (Prototype I), and 98.5 % ± 2.0 % (Prototype II) for centre A, see Fig. 5. For centre C it was 96.9 % (initial model), 96.9 % (Prototype I), and 96.4 % (Prototype II). The mean gamma value averaged over all the cases was similar for the initial model and the two prototypes at 0.34 ± 0.05 and 0.44 ± 0.1 for centre A and C respectively. Minor differences in the gamma index maps were seen as shown in the example given in Supplementary Figure S3. In the anthropomorphic plans of centre A, the difference between alanine measurements and TPS calculations were −0.34 % (initial), 0.37 % (Prototype I) and 1.15 % (Prototype II) for the SRS case, and 0.77 % (initial), 0.65 % (Prototype I) and 0.94 % (Prototype II) for the spine SBRT case. The uncertainty on the alanine measurement was 1.7 % at coverage factor k = 1. For centre C, the average dose difference for Farmer measurements over the 10 cases were −0.5 % ± 1.8 % (initial model), −0.1 % ± 1.3 % (Prototype I), and 0.2 % ± 1.4 % (Prototype II).

At centre B, plans B1, B3, B6, B11 and B13 were used for the tuning of MLC offset parameter in the prototypes. Both Prototype I and Prototype II improved the agreement in clinically relevant plans, with an increase in GPR of 2.3 % ± 0.8 % and 3.0 ± 0.8 %, respectively. The average GPRs were 93.1 % ± 2.3 % (initial model), 95.4 % ± 2.4 % (Prototype I), and 96.1 % ± 2.2 % (Prototype II). This translated to slight improvement in the gamma maps as shown in the example given in Supplementary Figure S4.

## 4 Discussion

This study has shown that it was possible to improve the modelling of an MLC with tilted leaf sided and rounded leaf tip within a commercial TPS. The aSG/SG tests provided a robust tool to tune TPS MLC transmission parameters, expose limitations in TPS MLC models and guide their improvement.

The initial MLC model could fit only part of the aSG curve, differences between centres were due to them arriving at different MLC parameters (Fig. 2 and Table 1). This agrees with previous publications reporting that the MLC parameters differ between centres [7], [18], [19], [20].

On the other hand, the two prototype MLC transmission models accurately replicated the measured SG/aSG doses with good fitting to the FOURL profile considering computation resolution, thus facilitating the standardisation of the configuration and commissioning processes. The small variation of the measured SG/aSG fields (Fig. 2) between centres indicated that it was sufficient to tune MLC transmission parameters to an average behaviour within each MLC class. Only the MLC offset parameter was adjusted (within ± 0.02 cm) for each machine and prototype, while the other parameters were kept constant. This can reduce the workload required for MLC configuration and its associated risks. It also allows for the establishment of clear references that are useful to reduce the variability in the parameters used by the community and to improve safety in clinical practice [5], [6], [21].

All MLC models achieved good accuracy in clinically relevant plans. Prototype I performed similarly to the more sophisticated Prototype II and constitutes a good option for implementation into TPSs. In centres A and C, the differences between prototypes and initial models were minor, while the prototypes outperformed their initial model at centre B (Fig. 5). This may be because in centre B the offset was adjusted based on PSQA results, which could be compensating for slight limitations in the device or its description within the TPS. However, this strategy might jeopardize the independence of PSQA devices and result in TPS configuration errors if systematic errors were present in such devices. We believe that adjusting the offset parameter based on ion chamber measurements of sweeping gap tests and clinically relevant plans is preferable and allows the use of PSQA devices as verification systems that are truly independent of the TPS configuration.

One limitation of this study is that only one commercial tilted MLC and the 6 MV energy was evaluated. We focused on the Eletka Agility because its modelling was shown to be particularly challenging and in need for improvement [7] and is used in many centres worldwide. However the same methodology could be applied to other present (such as the Elekta MLCi2 and Unity MLCs) and future tilted MLC to improve their modelling [9], [11]. Regarding the energy, it was already shown that the optimal MLC parameters for a given MLC type are quite independent of the nominal energy [9], but the presented procedure can be readily applied to other energies.

The fact that the two prototypes performed comparably for test fields and clinically relevant plans indicates that a highly sophisticated MLC model considering all the fine spatial details is not necessarily superior to a simpler MLC model when both models have been designed and fine-tuned to the aSG data. Hence, simple adjustments to existing MLC models can provide accurate calculations over a wide range of clinical plan characteristics and be easily configured using sweeping gap fields.

In conclusion, the aSG/SG tests provided a robust tool for MLC evaluation, comparison, and commissioning, and they expose limitations in MLC models and guide their improvement. It was possible to improve the modelling of a tilted MLC using simple adjustments to the existing transmission model. The similarity of the results between three different centers that validated the prototype models indicates that a common set of MLC parameters can be used, therefore reducing the workload required for this task and its associated risks, and improving the accuracy and safety of radiotherapy treatments.

## CRediT authorship contribution statement

**Mohammad Hussein:** Conceptualization, Methodology, Data curation, Formal analysis, Writing – original draft. **Agnes Angerud:** Conceptualization, Methodology, Software, Data curation, Formal analysis, Writing – review & editing. **Jordi Saez:** Conceptualization, Methodology, Formal analysis, Writing – review & editing. **Evelien Bogaert:** Data curation, Validation, Writing – review & editing. **Matthieu Lemire:** Data curation, Validation, Writing – review & editing. **Miriam Barry:** Data curation, Writing – review & editing. **Ileana Silvestre Patallo:** Data curation, Writing – review & editing. **David Shipley:** Data curation, Writing – review & editing. **Catharine H. Clark:** Supervision, Writing – review & editing. **Victor Hernandez:** Conceptualization, Methodology, Supervision, Writing – review & editing.

## Declaration of competing interest

The authors declare the following financial interests/personal relationships which may be considered as potential competing interests: NPL authors report institutional collaboration agreement with RaySearch Laboratories. Agnes Angerud is an employee of RaySearch Laboratories. The remaining authors have nothing to disclose.
