# Supplementary material for: Improving the modelling of a multi-leaf collimator with tilted leaf sides used in radiotherapy
Source: Phys Imaging Radiat Oncol. 2024 Feb 1;29:100543. doi: 10.1016/j.phro.2024.100543 (PMC10881418; doi:10.1016/j.phro.2024.100543)
Supplement: Supplementary data 1 [file mmc1.pdf]

Supporting Information

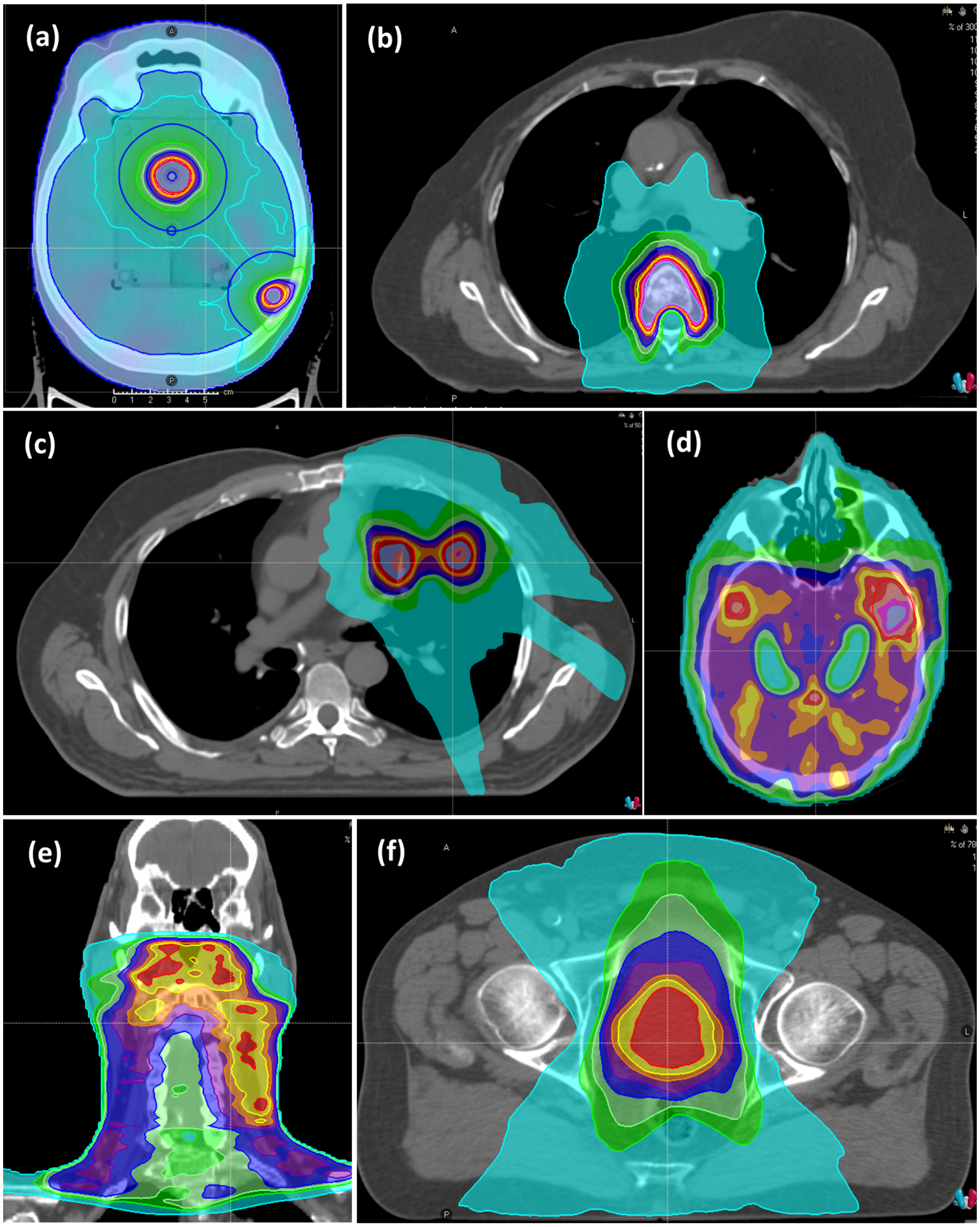

Figure S1: Examples of the variety of treatment plans used for the PSQA. (a) multiple metastasis stereotactic radiosurgery, (b) spine vertebra SBRT, (c) lung SBRT plan, (d) whole brain with hippocampal sparing, (e) head and neck, (f) prostate.

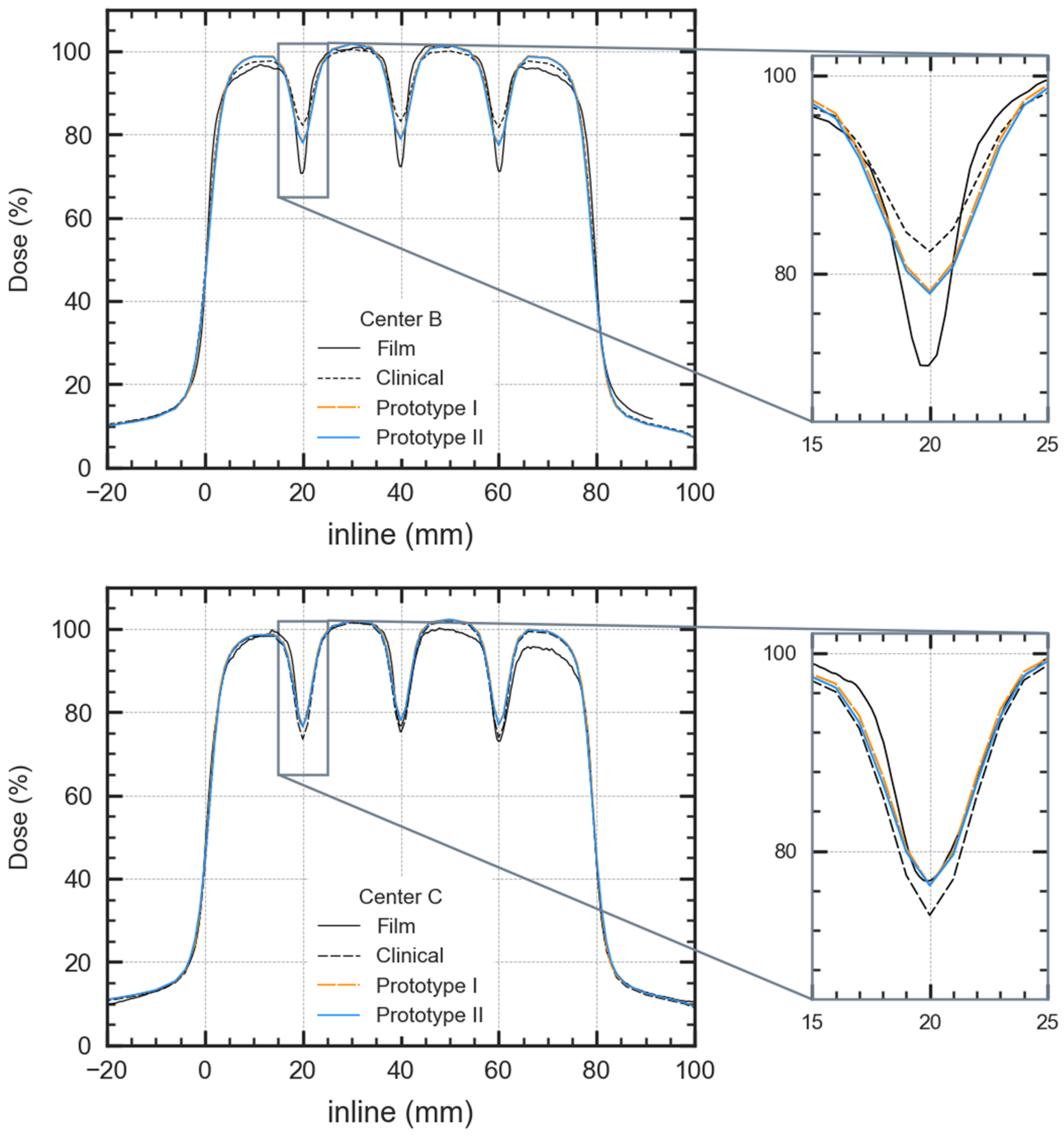

Figure S2: Line profiles extracted across the tongue-and-groove region of the FOURL from film measurements by center B and C and compared against the TPS dose calculations.

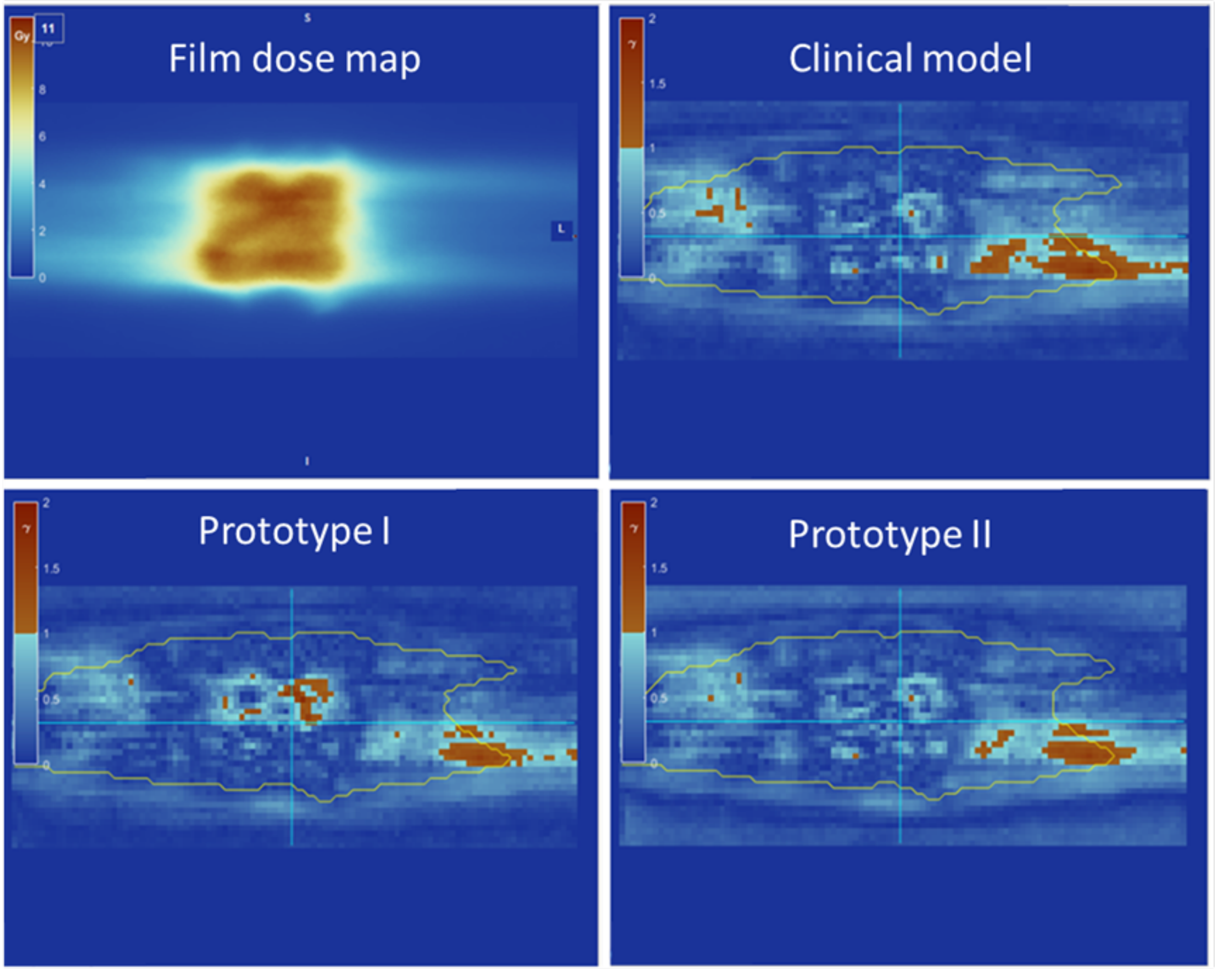

Figure S3: Example film PSQA result in an a modified CIRS Atom anthropomorphic phantom. 2%/2 mm γ maps for the spine SBRT vertebra case, with high MU to increase modulation. The film was measured in an axial plane through a tissue-equivalent vertebra at the T12 level.

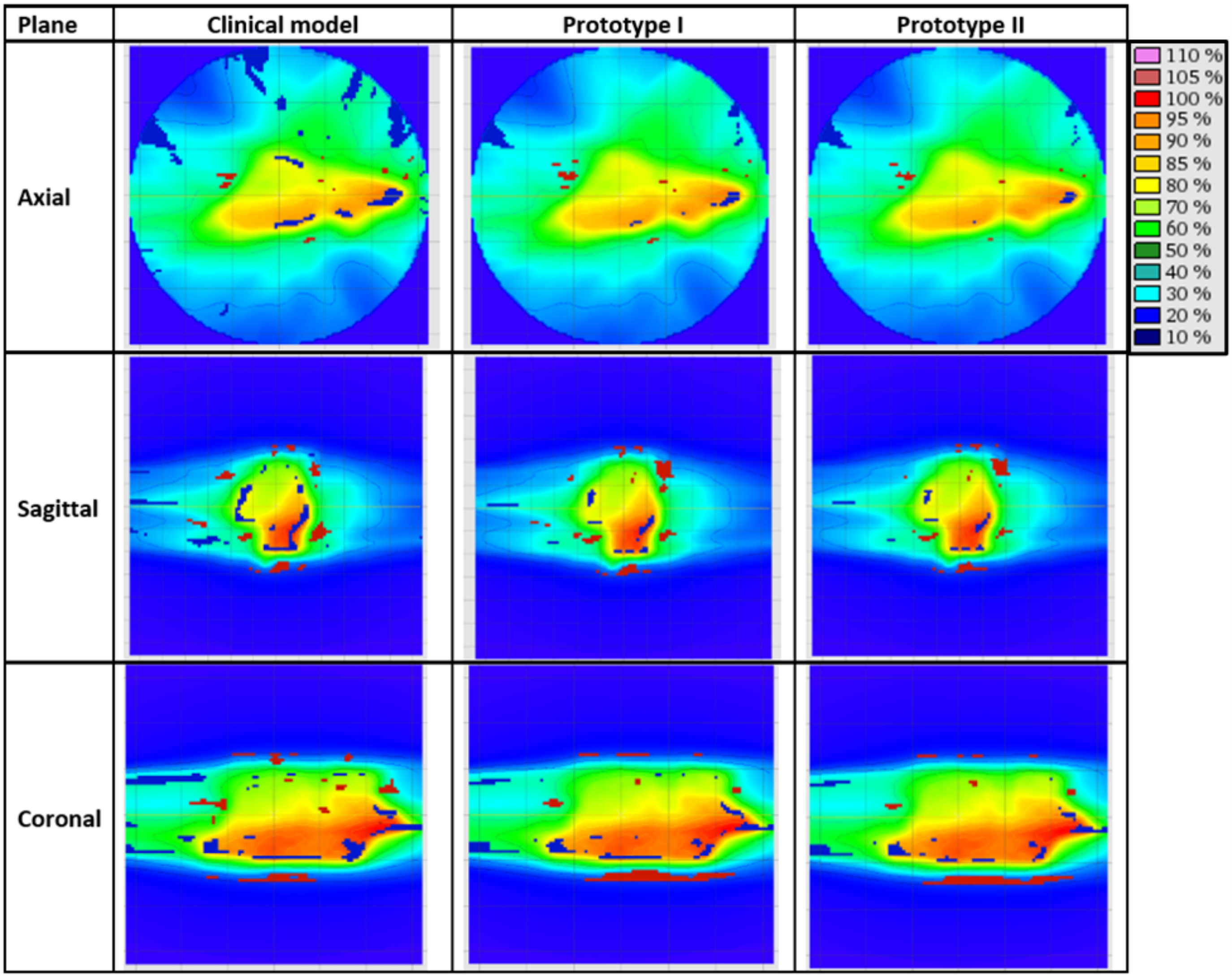

Figure S4: failed points of the gamma distribution of one of the esophagus cases measured by Centre B within the Octavius 4D phantom. Blue means cold (lower dose) and red hot (higher dose) with the respective plan doses as a reference. The isodose lines and color areas are depicted for the measured data with the % being relative to 1.8 Gy.
